# On the association between Chinese EFL teachers’ academic buoyancy, self-efficacy, and burnout

**DOI:** 10.3389/fpsyg.2022.947434

**Published:** 2022-07-22

**Authors:** Jie Ding, Li He

**Affiliations:** ^1^Department of College English Studies, Luoyang Normal University, Henan, China; ^2^Department of Languages and Communication Studies, Beijing Jiaotong University, Beijing, China

**Keywords:** teachers’ wellbeing, academic buoyancy, self-efficacy, burnout, Chinese EFL teachers

## Abstract

Buoyant and high self-efficacious teachers seem to be unlikely to feel burned out. Accordingly, examining the relationship between teachers’ buoyancy and self-efficacy seems significant. Even though the correlation between self-efficacy and burnout has been studied, precise attention should be paid to the quantitative investigations of the relationship between teachers’ academic buoyancy, self-efficacy, and burnout in China, English as a Foreign Language(EFL) context. To address this gap, the present study aimed to assess the relationship between Chinese EFL teachers’ academic buoyancy, self-efficacy, and burnout. In doing so, 399 Chinese EFL teachers (354 females and 45 males) were selected from 11 provinces and 18 cities in China. Three related questionnaires were virtually distributed among participants to gather data. The correlational tests revealed significant negative associations between Chinese EFL teachers’ burnout, self-efficacy, and academic buoyancy. The outcomes of multiple regression analyses also indicated that teachers’ burnout was negatively predicted by their self-efficacy and academic buoyancy. Finally, The implications of the results are discussed.

## Introduction

Academic buoyancy in L2 contexts is defined as the ability to negotiate and tackle the difficulties of education (Yun et al., 2018). Self-efficacy, as the second variable of this study, is conceptualized as a belief in the ability of individual teachers to plan, arrange, and apply the activities necessary to achieve a given educational goal (Skaalvik and Skaalvik, 2017; Fathi and Derakhshan, 2019; Fathi et al., 2020). Moreover, as the third variable of this study, burnout is defined as “a syndrome of emotional exhaustion, depersonalization, and reduced personal accomplishment” (Maslach and Jackson, 1986, p. 1).

Self-efficacy and buoyancy have something in common, which can both affect burnout, even though there are some discrepancies between these two constructs. Although the correlation between self-efficacy and burnout has been studied by several researchers (Skaalvik and Skaalvik, 2010; Motallebzadeh et al., 2014; Savas et al., 2014), to the researchers’ knowledge, the relationship between self-efficacy, buoyancy, and burnout has not been examined yet. Avanzi et al. (2013) reported a positive association between teacher self-efficacy and job satisfaction. Moreover, Malinen and Savolainen (2016) proposed that it is less probable for confident teachers to feel burned out. Another study conducted by Fathi et al. (2021) stressed the paramount role of teachers’ emotion regulation on their self-efficacy and burnout, meaning that those teachers that can control their emotions are more capable of feeling self-efficacious, and as a consequence, they are less likely to feel burned out. Furthermore, Skaalvik and Skaalvik (2009) also pinpointed that all the components of burnout and teacher autonomy and support are negatively correlated. In other words, teachers who do not feel burned out are more inclined to feel independent and more supportive of their students. It was also indicated by Lauerman and König (2016) that the association between self-efficacy, pedagogical knowledge, and burnout is negative. According to Etminan (2014), emotional fatigue and depersonalization were negatively correlated with work satisfaction, and personal achievement was positively correlated with work satisfaction. Moreover, Nayernia (2021) stated that language ability is negatively correlated with depersonalization and emotional exhaustion. Additionally, Zhaleh et al. (2018) in another study indicated that there was a significant link between intellect, tolerance of ambiguity, and the concept of burnout among teachers; therefore, teachers who reported feeling burned out were resistant to ambiguity.

It is of utmost importance to be noted that what caused the authors to consider the relationship among these three variables was the need to emphasize the contributory effect of academic buoyancy and self-efficacy in EFL teachers’ burnout since attention has recently been drawn to academic buoyancy, as a crucial factor which helps teachers tackle their work-related problems. What should be taken into consideration is that self-efficacy, academic buoyancy, and burnout can be studied together, which has hardly been done before since they together can cause teachers to come up with new ideas and achieve better results in terms of teaching methods and techniques.

Even though the correlation between self-efficacy and burnout has been studied, the quantitative investigations of the relationship between academic buoyancy, self-efficacy, and burnout in China, in an EFL context (English as a Foreign Language), have never been emphasized. From the perspective of teachers’ professional wellbeing, academic buoyancy is a factor that presents a combination of internal and external pressures on academic improvement and self-disciplinary attainment. The difficulties presented in education include teachers’ teaching skills and professional abilities, such as the mastery of information technology, which will give teachers pressure. However, self-efficacy is derived from teachers’ confidence in completing tasks with their own abilities and skills, which forms expectations and results from efficacy.

By focusing on the factors affecting teachers’ academic buoyancy and relating them to self-efficacy and burnout, the contradiction between teacher teaching and personal professional development can be described more comprehensively and dialectically. Thus, it provides constructive guidance for concrete teaching practice and teachers’ ability development. It can also help teachers correctly view the negative emotions and bottlenecks in the professional development of teachers and individuals and view the problems in education from the perspective of positive psychological and cognitive development to obtain a better professional identity.

Another point that should be emphasized is that although the relationship between teachers’ self-efficacy and burnout has been studied before, adding academic buoyancy provides more reliable results and a more productive study in education fields. Academic buoyancy that has recently been centralized in some studies in contrast to self-efficacy, which has been dealt with in prior studies, would add more weight to the current research.

To fill this gap, in this study, the attention has been focused on the association among these three variables, which can pave the way for both teachers and teacher educators to provide students with a more fruitful learning ambiance. One of the points that make this study seem of great significance is that both self-efficacy and academic buoyancy concentrate on a crucial fact that considering problems with which teachers are faced can be perceived as important, and the way these problems can be seen is what makes a difference among teachers and their teaching methods. Therefore, as these problems arise, teachers should pull themselves together and strive hard not to lose hope and see them as mood-boosters, leading to coming up with more practical solutions even though, these issues might be insoluble at first glance. Since many previous studies have focused on the relationship between teachers’ self-efficacy and their burnout (Han and Wang, 2021), the stressed point is that burnout which is regarded as destructive to the process of teaching, should be lessened by considering psychological capital, hence, it was the underlying reason behind conducting this study. Therefore, to bridge the gap in the literature, this research seeks to scrutinize the relationship between these variables. To this aim, the following research questions were posed:

• Are there any significant associations between Chinese EFL teachers’ academic buoyancy, self-efficacy, and burnout?
• Do academic buoyancy and self-efficacy significantly predict Chinese EFL teachers’ burnout?

## Literature review

### Academic buoyancy

Buoyancy is a psychological construct that deals with difficulties through a positive approach (Jahedizadeh et al., 2019). Academic buoyancy has been rooted in positive psychology, in which the critical role of emotions in the pedagogical domain is emphasized (Agudo, 2018). In such domains where problems can be found, buoyancy is conceptualized as the capability to cope with difficult situations with which one is faced (Martin and Marsh, 2020). As has been raised by Yun et al. (2018), in L2 contexts, it can be defined as the ability to negotiate and address the difficulties of learning and teaching a language. Both external and internal factors influence this construct. External factors are relevant to instructional contexts that are truly important to building interpersonal communication skills and academic buoyancy (Comerford et al., 2015).

In contrast, internal factors include characteristics such as being independent, motivated, self-efficacious, confident and having high self-esteem (Anderson et al., 2020). Some of the crucial principles which are associated with academic buoyancy go as follows; using strengths instead of focusing on weaknesses, challenges, and problems are addressed proactively rather than reactively as well (Martin and Marsh, 2020).

This term has been said to be relevant to some other terms, such as resilience, hardiness, immunity, and coping. These terms are different from each other to some extent. Even though they stem from the same theoretical ground, there is a discrepancy between them in that those problems which routinely happen in the individuals’ academic life are not clarified, considering resilience (Phan and Ngu, 2014). Moreover, it has been identified that the focus of resilience is on the difficulties of a small and extreme group of cases, whereas buoyancy concentrates on “many and healthy” cases caused by experiencing challenges in academic contexts (Martin and Marsh, 2020). Immunity, another synonymous term for buoyancy, refers to defensive mechanisms utilized to lessen the controversies, distractions, and damage by which one’s identity, motivation, and practice are affected (Hiver, 2017). Hardiness, another term that can be regarded as a synonym for buoyancy, is a characteristic that helps one fight and assuages stress’s effects on people (Hiver and Dörnyei, 2017). In other words, techniques that minimize the stress sources or modify the way people understand them should be practiced (Somerfield and McCrae, 2000). It should be highlighted that there have been many overlaps among the above-mentioned terms and more research should be conducted to clarify them.

In a study conducted by Yang et al. (2022), the impact of English language learners’ academic buoyancy and self-efficacy on L2 grit was studied. It was implied that language teachers had better focus on learners’ self-efficacy and academic buoyancy to help them in improving their L2 grit. It is more probable for students with higher buoyancy and efficacy to be gritty and to grow L2 grit. Efficacious and buoyant EFL learners should be encouraged by their teachers by attending to students’ emotional and motivational drives instead of merely cognitive ones. For the interests and efforts in language learning to be sustained, L2 learners need to consider the following factors: belief in personality, positively accepting their academic life, and being autonomous in coping with obstacles in learning L2.

One of the most crucial pillars of the educational system, regardless of the country, is teachers; therefore, their needs should be met to create a relaxed ambiance in which both teachers and learners feel highly motivated. That was why the association between teachers’ academic buoyancy and their self-efficacy and burnout was dealt with. On the other hand, academic buoyancy could be an essential factor that impacts many other constructs, such as the ones discussed in this study.

### Self-efficacy

Self-efficacy refers to belief in the actions necessary to achieve the intended goal (Bandura, 1997). This confidence in one’s ability to control and perform a set of measures to deal with subsequent events is also called self-efficacy (Bandura, 2006). Since then, the teacher’s self-efficacy can be conceptualized as the teacher’s self-confidence in their potential to manage the classroom situation to achieve the planned goals (Tschannen-Moran and Hoy, 2001). According to Skaalvik and Skaalvik (2017), self-efficacy is defined as teachers’ ability to plan, arrange, and apply the activities necessary to achieve a given educational goal. Thus, the teacher’s self-efficacy in this study refers to the teacher’s belief in the ability to organize and perform the set of actions necessary to successfully achieve a particular teaching task in a specific circumstance (Tschannen-Moran et al., 1998). Specifically, this construct is operationally conceptualized as an EFL teacher’s belief in the capability to apply educational strategies, maintain classroom leadership, and improve student involvement. As has been proposed by Tschannen-Moran and Hoy (2001), a teacher’s working life and his qualified education are equally dependent on three aspects of teacher effectiveness. These three aspects necessary for prosperous teaching comprise the application of teaching strategies, student involvement, and a sense of teacher effectiveness in using leadership techniques. Consequently, self-efficacious teachers accept new approaches, apply different educational strategies, and use management skills to increase student accompaniment and independence.

Avanzi et al. (2013) found a positive link between teacher self-efficacy and their satisfaction with their jobs. Malinen and Savolainen (2016) suggested that it is less probable for confident teachers to have burnout. Similarly, prior studies emphasized the significance of language teacher self-efficacy (Skaalvik and Skaalvik, 2007, 2010). In their qualitative research, Zonoubi et al. (2017) discovered the impact of Professional Learning Community (PLC) interventions on the self-efficacy of inexperienced and experienced EFL teachers. In addition, Cheon et al. (2018) pointed out that the effectiveness reached by intervention programs that support autonomy contributes to professional improvement. In another research study in Iran, Akbari and Allvar (2010) indicated that teacher personality traits, learners’ academic success, teacher self-efficacy, and ability to reflect were the best predictions of student performance. They attributed the teacher’s desire to develop self-efficacy to their enthusiasm for providing well-organized guidance. In other words, the positive emotions evoked by successful education can lead to teachers promoting self-efficacy (Tschannen-Moran and Hoy, 2007). Another study conducted by Fathi et al. (2021) stressed the paramount role of teachers’ emotion regulation on their self-efficacy and burnout which means that those teachers that can channel their emotions are more capable of feeling self-efficacious, and as a result, they are less likely to feel burned out.

### Burnout

Long-term work stress under which a person puts is regarded as a psychological syndrome called burnout (Maslach, 2003). This term was first coined by Freudenberger (1974). To work too much without paying attention to someone’s needs is regarded as a state of exhaustion (Byrne, 1999). Then, burnout was conceptualized as “a syndrome of emotional exhaustion, depersonalization, and reduced personal accomplishment that can occur among individuals who do “people work” of some kind” by Maslach and Jackson (1986, p. 1). However, according to Pines and Aronson (1988), emotional, mental, and physical exhaustion are caused by prolonged exposure to emotionally demanding situations. Burnout is also defined as the effort to overcome the work-related stress one has been under for a long time and fails to succeed (Jennett et al., 2003). Concerning teachers’ burnout, emotional exhaustion can be defined as energy depletion when one is emotionally exhausted.

Similarly, depersonalization, another dimension of burnout, is when teachers feel callous and are not fond of their students and their job anymore. Reduced personal achievement is associated with not feeling competent enough and not teaching effectively to help their students throughout the learning process (Maslach et al., 2001). Both individual and environmental factors are the underlying causes of burnout. Skaalvik and Skaalvik (2010) indicated an association between burnout, self-efficacy, job satisfaction, and context-related factors. It was also shown that the time pressure teachers face and the teacher-parent relationship were the critical predictors of depersonalization and emotional exhaustion. Likewise, although the association is weak, discipline-relevant problems about students’ behavior and emotional depletion have been identified to be significantly correlated with depersonalization.

Skaalvik and Skaalvik (2009) also pinpointed that all the subscales of burnout and teacher autonomy and support are negatively correlated. In other words, teachers who do not feel burnout are more inclined to feel independent and more supportive of their students. Another research conducted by Ghanizadeh and Ghonsooly (2014) showed that institutional supervision is a predictor of teachers’ burnout, meaning that being monitored and supervised by institute authorities causes teachers to feel burned out. Furthermore, it was indicated by them that there was a negative and significant correlation between self-regulation and teacher burnout. It was also disclosed by Lauerman and König (2016) that the association between self-efficacy, pedagogical knowledge, and burnout is negative.

Another study by Eghteasadi Rudi (2011) pointed out that poor student proficiency and lack of government support are the main reasons for teacher burnout. In addition, autonomous, self-efficacious, and extroverted students resisted burnout compared to classmates with lower levels of personal traits. In addition, work satisfaction was found to play an essential role in all three burnout subscales (Etminan, 2014). In other words, emotional fatigue and depersonalization were negatively correlated with work satisfaction, and personal achievement was positively correlated with work satisfaction. Nayernia (2021) stated that language ability is negatively correlated with depersonalization and emotional exhaustion. Based on Faskhodi and Siyyari (2018), there was a significant negative association between work engagement and burnout. In addition, it has been reported that burnout decreases with the increasing duration of the experience. In contrast, the teacher’s experience is positively correlated with work involvement.

Furthermore, independent, self-efficacious, and outgoing teachers are resistant to burnout in contrast to their counterparts with lower levels of the mentioned characteristics. Furthermore, job satisfaction is one of the paramount factors impacting all the dimensions of burnout (Etminan, 2014). There was a negative relationship between emotional exhaustion, depersonalization, and job satisfaction, while there was a positive correlation between personal accomplishments and job satisfaction. Nayernia (2021) reported that language proficiency is negatively correlated with depersonalization and emotional exhaustion as two dimensions of burnout, and it is positively in line with personal achievement that is another dimension of burnout. Faskhodi and Siyyari (2018) found a significant and negative relationship between work engagement and burnout.

Zhaleh et al. (2018), in another study, indicated that there was a significant relationship between intellect, tolerance of ambiguity, and the concept of burnout among teachers; therefore, teachers who reported feeling burned out were resistant to ambiguity.

In addition, emotion is one of the concepts that may be associated with burnout. Emotionality is a mixture of emotional and sensory frequencies, which means that sensually evoked emotions can relativize perception. Depending on emotions, an individual can develop (listen to and see something) and be involved (experience something directly). It seems that emotional levels can affect burnout. The higher the emotion, the less likely it is to have burnout (Pishghadam et al., 2016). Accordingly, the relationship between emotions and burnout can be discussed in future studies.

As can be seen, burnout can adversely affects several components such as emotional intelligence, motivation, and wellbeing. However, burnout plays a positive role in people’s performance and success because when they are emotionally exhausted. They struggle to find a solution to the so-called insoluble problems, which can significantly increase their creativity. No studies have been done, particularly in China, probing the association between these three variables. Therefore, This research aims to discover the associations between teachers’ academic buoyancy, self-efficacy, and burnout. As emphasized in this study, the focus is on highlighting the impact of EFL teachers’ academic buoyancy on teachers’ self-efficacy and their burnout. The studies mentioned above mainly concentrated on the relationship between teachers’ self-efficacy and burnout even though these days, academic buoyancy is what has attracted attention and can tremendously affect both self-efficacy and burnout, Despite the fact that it has been confirmed in some studies that self-efficacy and burnout are negatively correlated, the opposite result might be taken into consideration as well; in that when teachers feel emotionally exhausted (burnout), they endeavor to tackle a problem they face, and it can ameliorate their creativity to a great extent to find a solution to the problem. Therefore, it can also be perceived as positive rather than negative. As a result, the relationship between self-efficacy and academic buoyancy might be positive, which can be studied in further research in the future.

## Materials and methods

### Participants

To gather a large sample, the convenience sample selection *via* Wejuanxing software was adopted, a popular and commonly used data collection tool in China. Because of the COVID-19 pandemic, we distributed our questionnaire *via* WeChat to collect valid data. After our data collection, 399 Chinese EFL teachers were randomly selected from 11 provinces and 18 cities in China. The sample included 354 females (88.72%) and 45 males (11.28%), ranging in age from 21 to 68. They were from various levels of teaching (24.81% primary school, 24.56% middle school, 10.28% high middle school, 40.34% college). Their teaching experience ranged from 1 to 41 years. Since there exists a preference tendency for female students concerning the teaching experience, the sample can be classified into three groups: novice teachers (34.27%), experienced teachers (25.32%), and highly experienced teachers (40.41%). The participants were also asked to report their majors and education levels. The detailed demographic information is presented in Table 1.

**TABLE 1:** Demographic information of the participants.

Demographic information category	N	%
**Gender**		
Male	45	11.28
Female	354	88.72
Total	399	100
**Age**		
21–29	139	35.1
30–39	98	24.7
40–49	131	33.1
50–59	24	6
60–68	4	1
Total (valid)	396	100
Missing cases	3	
Total	399	
**Level of education**		
High school level	2	0.56
Bachelor’s degree	205	57.42
Master’s degree	136	38.1
Doctoral degree	14	3.92
Total (valid)	357	100
Missing cases	42	
Total	399	
**Major**		
English and literature	19	4.76
Foreign linguistics and applied linguistics	294	73.68
Education	58	14.54
Others	28	7.02
Total	399	100
**Teaching experience (year)**		
0.1–4	134	34.27
5–15	99	25.32
16–42	158	40.41
Total (valid)	391	100
Missing cases	8	
Total	399	

### Instruments

Teacher self-efficacy was assessed using the 24-item scale designed by Woolfolk et al. (1990). The items were presented in a 5-Likert scale format in which the teachers selected a number to indicate their opinion on each item (1 Not high to 5 A great deal). The Cronbach alpha test was run to make sure of the reliability of the questionnaire administered in this study. It was indicated that the teacher burnout scale (0.96) had satisfactory reliability indices. Academic buoyancy was evaluated utilizing the ABS (Academic Buoyancy Scale) with four items developed by Martin and Marsh (2008). The teachers answered the questionnaire.

This was a seven-point scale (from 1 “strongly disagree” to 7 “strongly agree”). Items were supposed to operationalize the controversies and setbacks teachers might face as a matter of course at school. This scale (0.81) in the present study had satisfactory reliability indices. For teachers’ burnout to be measured, the teacher version of the Maslach burnout inventory (MBI-ES), validated and piloted by Maslach et al. (1997), was utilized to measure the burnout of participant instructors in the current study. MBI-ES comprises 22 items that evaluate the following subscales: Burnout, reduced personal achievement, depersonalization, and emotional exhaustion. Each item of this questionnaire is rated on a seven-point Likert scale ranging from 0 (never) to 6 (every day). The Cronbach alpha test was run to ensure the reliability of the questionnaire used in this study. It was shown that the teacher burnout scale (0.85) had satisfactory reliability indices.

### Data collection procedure

Approval for data collection was obtained from the school authority before conducting the research. The purpose of the questionnaire was explained to teachers who were involved. Participants were voluntary and informed that they could refuse or reject any unpleasant questions according to their willingness. More importantly, participants were told that their data would only be used for research purposes under the premise of anonymity. All items in the questionnaire were administered in bilingual style (Chinese and English) to avoid the mismatch between ambiguity and translation. The questionnaire was created through a well-known Chinese online website for designing and distributing the questionnaire, whose Chinese name is Wenjunxing. The QR code of this questionnaire was shared on WeChat. The teachers completed the questionnaires on their own time through WeChat on mobile phones or computers. Participants were also given detailed instructions on what the following scale measures and how to respond to each item. The data collection took more than 3 months, from November 2021 to February 2022. All the 399 participants submitted their responses in the end. They provided demographic information as shown in the above table and completed all the questionnaires.

### Data analysis

Spearman Rho was used in this study to find the relationship between self-efficacy, teacher burnout, and academic buoyancy. Additionally, multiple regression was utilized to assess the statistical significance of the results.

## Results

The first step in analyzing the data is to measure the reliability of the instruments used to gather the data. The reliability indices were calculated by running Cronbach alpha.

It was indicated that the self-efficacy questionnaire (0.96), teacher burnout questionnaire (0.85), and academic buoyancy questionnaire (0.81) had satisfactory reliability indices. A normality test was run to decide upon the parametric or non-parametric analysis. The results are shown in the following:

The indices of Kolmogorov-Smirnov (Table 2) show that the distribution of data was not typical for any of the variables since the *p*-value is lower than the significance level (*p* < 0.05). Consequently, the non-parametric analysis, the Spearman Rho test, was used.

**TABLE 2:** Test of normality.

	Kolmogorov-Smirnov^a^			Shapiro-Wilk		
	Statistic	df	Sig.	Statistic	df	Sig.
AB	0.064	399	0.000	0.989	399	0.004
SE	0.057	399	0.004	0.988	399	0.002
BO	0.045	399	0.049	0.994	399	0.096

^a^Lilliefors significance correction.

### The first research question

The first research question deals with the relationship among three variables of this study (i.e., self-efficacy, teacher burnout, and academic buoyancy) which was calculated by running a Spearman Rho correlation test.

Table 3 shows the relationship between the Chinese EFL teachers’ self-efficacy, their burnout, and academic buoyancy. This table shows negative (–0.19, –0.27) and significant (sig = 0.00, and 0.04) relationships between teachers’ burnout, their academic buoyancy, and self-efficacy. It can be concluded that if teachers’ index of burnout increases, the indices of teachers’ buoyancy and self-efficacy decrease.

**TABLE 3:** Correlations between self-efficacy, teacher burnout, and academic buoyancy.

			AB	SE
Spearman Rho	BO	r	–0.192	–0.272
		Sig. (2-tailed)	0.006	0.041
		N	399	399

### The second research question

The second research question measures the prediction power of Chinese EFL teachers’ self-efficacy and buoyancy for their burnout. To this end, a linear multiple regression analysis was performed in the following tables.

The model summary (Table 4) indicates how much of the variance in the dependent variable [scores obtained from the dependent variable (teachers’ burnout)] can be explicated by the model (which comprised the following variables: self-efficacy and academic buoyancy). Expressed as a percentage, it implies that the model explained 33.5 percent of the variance in scores from teachers’ burnout. The value was 0.48 (*R*^2^ = 0.335).

**TABLE 4:** Model summary for teachers’ self-efficacy, academic buoyancy, and burnout.

Model	*R*	*R* ^2^	Adjusted R square	Std. The error in the estimate
1	0.487	0.335	0.030	21.37

Dependent variable: Burnout.

Predictors: (Constant), self-efficacy, academic buoyancy.

To assess the statistical significance of the results, it was necessary to look at Table 5 labeled ANOVA. This tested the hypothesis that multiple R in the population equals zero (0). The model reached statistical significance [*F* = (2, 396) = 7.19, Sig = 0.000, this really means *p* < 0.05].

**TABLE 5:** ANOVA for teachers’ self-efficacy, academic buoyancy, and burnout.

Model		Sum of squares	Df	Mean square	*F*	Sig.
1	Regression	6573.62	2	3286.81	7.19	0.000
	Residual	180871.79	396	456.74		
	Total	187445.41	398			

Dependent Variable: Burnout.

Predictors: (Constant), self-efficacy, academic buoyancy.

In this research, it was the researchers’ interest to compare the contribution of each independent variable; thus, they utilized the beta values. Considering the Beta column, Table 6 reported that self-efficacy accounted for the most significant beta coefficient 0.19 (sig = 0.000), meaning that this variable has made the most decisive contribution to clarifying the dependent variable when the variance explicated by all other variables in the model was controlled. The Beta value (0.13) for the other variable (i.e., academic buoyancy) was also significant (sig = 0.01).

**TABLE 6:** Coefficients for teachers’ self-efficacy, academic buoyancy, and burnout.

Model		Unstandardized coefficients		Standardized coefficients	*t*	Sig.
		B	Std. error	Beta		
1	(Constant)	66.33	6.19		10.70	0.000
	AB	–0.55	0.22	–0.13	–2.45	0.015
	SE	–0.31	0.08	–0.19	3.63	0.000

Dependent variable: Burnout.

## Discussion

This study is aimed at scrutinizing the relationship between Chinese EFL teachers’ academic buoyancy, self-efficacy, and burnout. The current research findings indicated a positive correlation between teachers’ academic buoyancy and their self-efficacy, while the association between teachers’ academic buoyancy and their burnout was negative. To justify the results, it should be said that buoyant teachers, who are claimed to overcome the educational difficulties, are highly likely to be self-efficacious and unlikely to feel burned out. Hence, rarely do they lose their hope of teaching. Moreover, finding a solution to the problems with which they are faced is what such teachers mostly consider. This finding is partly compatible with Han and Wang’s (2021) study, which also confirmed a positive correlation between Chinese EFL teachers’ academic engagement and their self-efficacy. Buoyancy, which is the ability to negotiate and address the difficulties of learning and teaching a language, was aligned with self-efficacy. Therefore, it is more probable for self-efficacious teachers to be buoyant. Such teachers do not find it hard to challenge themselves whenever they get stuck in difficult situations to think of new solutions that might not have been practiced before. Emphasis should be put on the fact that happiness and anguish are interwoven even in the learning and teaching contexts. Burnout as anguish seems to pave the way through feeling buoyant and self-efficacious sometimes; hence, analyzing burnout from this aspect and its impact on self-efficacy and buoyancy is the title that should be further studied.

Buoyant teachers deal with schoolwork pressures and do not allow anything to lower their confidence once it has been built. Moreover, negative feedback is not considered as setbacks since they show teachers how to channel their emotions in academic contexts. As a result, setbacks that turn out to be one of the worst experiences that happened to teachers are overcome by buoyant teachers (Wang and Guan, 2020). Regarding self-efficacy, how much a teacher can do to get through the problematic students is said to be relevant to self-efficacious teachers. Another feature for teachers with higher self-efficacy is helping students feel motivated to be actively involved in-class activities, especially those who express low interest. Students should be taught to think critically and always see the issues from a different aspect, they should be taught to value learning, and their creativity can be fostered through giving them meaningful tasks by which they are challenged.

On the other hand, teachers who feel burned out are emotionally exhausted due to work, and they are not fond of what is going on with their colleagues, so it is the reason why they cannot establish a relaxed atmosphere in their working environment. Regarding the points, these teachers cannot cause students to feel motivated when failing or allow them to learn to think critically because they are not full of energy themselves. They cannot also give students an insight to see the difficulties they face as a stepping stone to their success, even though they feel tired when they get up in the morning and see a new working day stretched out in front of them.

It should be highlighted that making progress and feel content during the language learning process are associated with positive emotions (Dewaele and Macintyre, 2016; Li, 2020). Furthermore, many studies have concentrated their attention on emotions that can pose meaningful contributions to L2 learning (Li et al., 2020; Wang and Guan, 2020). Although buoyancy and self-efficacy are not like enjoyment and love to be regarded as positive emotions, they both have their positive impacts on the educational contexts. For instance, when teachers are both buoyant and self-efficacious, it is more probable for them to address the problems they face through the learning process, and they may feel glad after their problems have been solved. As proposed by Gregersen (2013), the process of learning is facilitated by positive psychology and allows the EFL learners and teachers to enjoy it more; hence, when teachers feel less burned out and more buoyant, it seems to cause the facilitation in the process of learning and such a learning atmosphere, learners are more liable to be inclined to learn new materials. Buoyancy can be of pivotal significance in this study because it can keep teachers’ spirits up, leading to a better context for teaching and being creative to make the atmosphere of the class more productive and interactive.

This study is in line with the following studies due to the reasons which are going to be discussed below (Maslach et al., 2001; Tschannen-Moran and Hoy, 2001; Akbari and Allvar, 2010; Comerford et al., 2015; Lauerman and König, 2016; Malinen and Savolainen, 2016; Martin and Marsh, 2020). As mentioned by Comerford et al. (2015), academic buoyancy and interpersonal skills are believed to be associated with external factors, particularly in instructional circumstances. It is relevant to the findings of this study because the results evidenced that both buoyancy and self-efficacy, which can be perceived as interpersonal factors, are negatively correlated with burnout. Both buoyant and self-efficacious teachers who try to relieve the stress caused by educational problems are less likely to experience burnout. Moreover, the results achieved in the current study are in line with what has been proposed by Martin and Marsh (2020). Burnout harms academic buoyancy and does not allow the strengths to be emphasized. As a result, teachers show reactive reactions toward problems. The results of the present study are also in congruence with what has been shown by Tschannen-Moran and Hoy (2001). In that self-efficacious teachers are perceived to be self-confident in managing their class and channeling their feelings when facing a difficult situation to reach their planned goals; therefore, self-efficacy has a negative relationship with burnout whose components are depersonalization and emotional exhaustion and reduced personal achievement.

Similarly, these results lend support to another study conducted by Malinen and Savolainen (2016) since confident teachers with higher levels of self-efficiency are less prone to feel burned out. The findings are also in line with the study carried out by Akbari and Allvar (2010) in that it was explained students’ performance and academic success are affected by teachers’ personality traits. Hence, in this respect, buoyancy and self-efficacy are significantly and negatively correlated with burnout. The results also give credence to a study conducted by Lauerman and König (2016), who justified a negative relationship between self-efficacy, pedagogical knowledge, and burnout, since those teachers who experience burnout are less likely to boost their knowledge and see the problems as stepping stones to their success.

The present study’s findings also support those of the study conducted by Yang et al. (2022), even though the emphasis was placed on EFL students rather than teachers in the referred study. The two components mentioned above have one thing in common, they both cause teachers to overcome their obstacles. In the current study, it has been confirmed that buoyant, self-efficacious teachers are less likely to experience burnout. However, in the one done by Yang, it was highlighted that both EFL students’ self-efficacy and academic buoyancy were indicative factors for students’ grit and the amount of hard work that can be put into practice to make things happen. Moreover, the present study’s findings also give credence to that of Fathi et al. (2021), in which the pivotal role of emotion regulation in teachers’ self-efficacy and burnout was emphasized. When regulating emotions, teachers are highly likely not to feel burned out and to feel efficacious. Both emotion regulation and academic buoyancy can act as mediators for teachers’ self-efficacy and burnout.

## Conclusion

This research was conducted to scrutinize the relationship between teachers’ self-efficacy and their academic buoyancy and burnout among Chinese EFL teachers. It was reported that teachers’ burnout is negatively and significantly associated with teachers’ self-efficacy and their academic buoyancy. As a result, it should be highlighted that teachers’ burnout can predict teachers’ self-efficacy and their academic buoyancy. The results of this study seem to be significant for teacher educators, educational authorities, and EFL teachers.

This research can be of paramount importance for EFL teachers, students, and educational authorities. Educational authorities should put all their effort into practice so as not to allow teachers to feel burned out. As known, prevention is better than cure; hence, teachers should be provided with strategies to learn how to feel buoyant and self-efficacious, contributing to not experiencing burnout. There should be some psychologist with whom teachers can talk about their problems, through which their minds are challenged and they can empty themselves since sometimes these are the pent-up emotions that do not allow teachers to act appropriately when teaching or being prepared for classes. On the other hand, students can benefit from this study as well since teachers way of treatment influences students’ academic achievement, productivity, enthusiasm for learning, and success. Not only do they have the right to have buoyant teachers who see the glass as half complete, but they also need to learn how to act in this way and be independently faced with education-related problems. A significant part of students’ worldview has been shaped while they are involved with learning, and they can experience a joyful life in their future. It is no denying that when teachers are in good health and enjoy both mental and physical wellbeing, they are highly likely to be committed to their jobs and strive hard to get a promotion. Therefore, the educational infrastructures will be strengthened by those teachers who feel committed to what they do. Because the way they come up with new ideas can change their teaching methods, EFL students feel motivated enough to increase their knowledge of the language and be actively engaged in the classroom activities in which real-life exercises are practiced.

This research has its own limitations. First, it is limited to China, where this study was conducted. Without a doubt, the results might be different from region to region since it is not culture-specific. In addition, there exist gender unbalances in the research participants. In the Chinese educational context, there are still some preferences for female students who would like a secure professional prospect, which will lead to the number of female teachers outweighing their counterparts. Therefore, the large proportion of samples in the questionnaires are female teachers. Given this fact, further studies can be conducted concerning the above-mentioned factors in other similar or dissimilar educational contexts. Different aspects of participants, including gender, age, and differences in educational subjects, can also be further examined. Thirdly, although quantitative studies are reliable since they are objective, longitudinal studies can be conducted in the future to have more in-depth ideas about it. Finally, even though the interaction among these three variables has not been studied before, academic buoyancy, a novel concept, can be studied using some other variables in future research by avid researchers. The relationship between the variables can still be further deepened in statistical methods, such as using a structural equation model.

## Data availability statement

The raw data supporting the conclusions of this article will be made available by the authors, without undue reservation.

## Ethics statement

The studies involving human participants were reviewed and approved by the Luoyang Normal University Ethics Committee. The patients/participants provided their written informed consent to participate in this study.

## Author contributions

Both authors listed have made a substantial, direct, and intellectual contribution to the work, and approved it for publication.

## Conflict of interest

The authors declare that the research was conducted in the absence of any commercial or financial relationships that could be construed as a potential conflict of interest.

## Publisher’s note

All claims expressed in this article are solely those of the authors and do not necessarily represent those of their affiliated organizations, or those of the publisher, the editors and the reviewers. Any product that may be evaluated in this article, or claim that may be made by its manufacturer, is not guaranteed or endorsed by the publisher.
